# Indocyanine Green Loaded Polymeric Nanoparticles: Physicochemical Characterization and Interaction Studies with Caco-2 Cell Line by Light and Transmission Electron Microscopy

**DOI:** 10.3390/nano10010133

**Published:** 2020-01-11

**Authors:** Antonella Obinu, Elisabetta Gavini, Giovanna Rassu, Federica Riva, Alberto Calligaro, Maria Cristina Bonferoni, Marcello Maestri, Paolo Giunchedi

**Affiliations:** 1 Department of Chemistry and Pharmacy, University of Sassari, 07100 Sassari, Italy; obinuantonella@gmail.com (A.O.); grassu@uniss.it (G.R.); 2 Department of Public Health, Experimental and Forensic Medicine-Histology and Embryology Unit, University of Pavia, 27100 Pavia, Italy; federica.riva01@unipv.it (F.R.); alberto.calligaro@unipv.it (A.C.); 3 Department of Drug Sciences, University of Pavia, 27100 Pavia, Italy; mariacristina.bonferoni@unipv.it; 4 IRCCS Policlinico San Matteo Foundation and Department of Clinical-Surgical, Diagnostic and Paediatric Sciences, University of Pavia, 27100 Pavia, Italy; mmaestri@smatteo.pv.it

**Keywords:** indocyanine green, polymeric nanoparticles, fluorescence imaging, indocyanine green stability, tumor treatment and detection

## Abstract

Biomedical applications of nanoparticles (NPs) have reached an increasing development in recent years. Recently, we demonstrated that newly synthesized poly (ethyl 2-cyanoacrylate) nanoparticles (PECA-NPs) are possible antitumor agents due to their cytotoxicity for cancer cells. Indocyanine green (ICG), an amphiphilic tricarbocyanine fluorescent dye, is widely used for the detection of tumoral extension in different organs during clinical surgery. Moreover, this fluorescent agent is unstable and it has a rapid clearance in physiological conditions in vivo. In this study, ICG was charged in PECA-NPs to improve its aqueous stability and make easier its use for the identification of tumor cells. Microscopic and ultrastructural aspects concerning the related in vitro interactions between ICG-loaded NPs and tumor cell culture were investigated. Obtained results showed an effective stabilization of ICG; furthermore, color inclusions inside the cells treated with ICG-loaded NPs demonstrated the internalization of NPs with associated ICG. Transmission electron microscopy (TEM) analysis demonstrated the cytoplasmic presence of coated vesicles (Ø ≤ 100 nm), hypothesizing their involvement in the mechanism of endocytosis. Therefore, ICG-loaded NPs could be proposed as agents for tumor diagnosis, hypothesizing also in the future a specific therapeutic treatment.

## 1. Introduction

Poly (alkyl cyanoacrylate) nanoparticles (NPs) have been widely investigated due to their biodegradability, biocompatibility, favorable stability, and for their potential in cancer treatment [1,2]. Recently we described the preparation of poly (ethyl 2-cyanoacrylate) nanoparticles (PECA-NPs) using an emulsion polymerization method. PECA-NPs physicochemical properties were assessed and their ability to support anticancer activity on 3D tumor models of hepatocellular carcinoma and kidney adenocarcinoma was demonstrated [3]. This work confirms what was previously pointed out for PECA-NPs by other authors that proposed them as a carrier for cancer treatment, showing capabilities to target and control drug delivery [4]. On the basis of these results, we decided to study the loading of PECA-NPs with a fluorescent dye to improve the usefulness of previously prepared NPs in cancer management.

Indocyanine green (ICG) was selected as a fluorescent marker. It is an amphiphilic tricarbocyanine dye characterized by absorption and emission bands in the near-infrared (NIR) region. When exposed to an excitation light between 750 and 810 nm, ICG exhibits fluorescence at 840 nm [5]. It is the only NIR dye approved by the United States Food and Drug Administration in clinical use for medical diagnosis and imaging [6]. Thanks to its fluorescence ability, ICG has been extensively used for over 50 years as an agent for the evaluation of liver function, cardiac output, pharmacokinetic analysis, ophthalmic angiography, and visualization of retinal and choroidal vasculatures [7,8]. Among its positive characteristics, it enables deep penetration into tissues, low light scattering, and tissue autofluorescence [9]. Currently, ICG is widely employed in tumor precision image-guided surgery. Several authors reported the use of ICG to improve the intraoperative detection of neoplastic lesions and in sentinel lymph node mapping [10,11,12]. This allows the surgeon to more precisely identify the tumor margins with respect to healthy tissue, improving intervention precision and complete removal in shorter times [13,14]. This approach attracted particular attention in the hepatic tumor interventional oncology both by laparoscopic and open surgery [15,16,17]. However, despite the numerous benefits, the clinical application of ICG has some limitations. ICG easily binds in a non-specific manner to plasma proteins after intravenous administration, and it is subject to a rapid clearance (plasmatic half-life of around 2–4 min) mainly via the activity of the reticuloendothelial system (RES) [18]. In addition, ICG is very unstable both in vitro and in vivo because of its aqueous instability, thermal degradation, and photo degradation [19,20]. In aqueous solutions, ICG is affected by physicochemical changes like irreversible degradation and time-dependent aggregation, resulting in complete precipitation in about 24 h, with reduced fluorescence and discoloration [21]. In order to overcome these limitations, the loading of ICG into various carriers has been proposed [22,23,24,25]; particularly, the NPs have received great interest as promising ICG delivery systems [20,26,27,28].

NP systems present unique advantages in nanodiagnostics, where their properties support not only specific biodistribution but also improve signal density and contrast [29]. More recently, NPs have been proposed for their capacity to merge therapeutic and diagnostic approaches within a single formulation, demonstrating a potential for theranostic purpose, and can be considered greatly useful in the emerging field of personalized medicine [30,31,32].

Thus, the aim of this work was the development of a nanosystem able to improve the fluorescence properties of ICG, and therefore its use in tumor diagnosis, combining them with anticancer activity of the PECA-NPs already determined. The loading of PECA-NPs with a fluorescent dye allows us to investigate the interaction of NPs themselves with a cancer cell-substrate. The human colorectal adenocarcinoma cell line (Caco-2) is a good epithelial intestinal tumor model with an epithelioid phenotype, usually used to study the absorption and uptake mechanisms and also used to assess the cytotoxicity by MTT assay [33]. The internalization of the loaded systems was tested in vitro into Caco-2 cell cultures as a model of colon cancer and of metastatic colon cancer lesions in the liver. PECA-NPs loaded with ICG were studied in comparison to free ICG alone.

Furthermore, during this study, we evaluated if the dye-loading process could improve the aqueous stability of ICG. This could offer the possibility to prolong ICG visualization that might be particularly useful during the surgical resection of the tumor. Therefore, ICG-loaded NPs were characterized in terms of size and fluorescence properties, with special attention to the stabilization effect of ICG resulting from its association with PECA-NPs.

## 2. Materials and Methods

### 2.1. Materials and Reagents

Ethyl 2-cyanoacrylate (ECA) was used as a monomer for the polymerization. Cardiogreen^®^ (ICG) and Tween 20 were obtained from Sigma-Aldrich (St. Louis, MO, USA). Phosphotungstic acid was purchased from Carlo Erba reagents s.r.l. (Milan, Italy). Antibiotic/antimycotic solution (100×), containing 10,000 units/mL penicillin, 10 mg/mL streptomycin, 25 mg/mL amphotericin B, and dimethyl sulfoxide (DMSO), were purchased from Sigma-Aldrich (Milan, Italy). Dulbecco’s Modified Eagle Medium (DMEM with 4.5 g/L glucose, l-glutamine, and sodium pyruvate) was purchased from Corning (Mediatech Inc. A Corning Subsidiary Manassas, Manassas, VA, USA). Dulbecco’s Phosphate Buffer Solution and inactivated fetal calf serum were acquired from Biowest (Nuaillé, France). MTT (3-(4,5-dimethylthiazol-2-yl)-2,5-diphenyltetrazolium bromide) and trypan blue solution was purchased from Sigma-Aldrich (Milan, Italy). Cell line culture Caco-2 was obtained from the European Tissue Culture Collection. Ultrapure bi-distilled water was obtained by a MilliQ R4 system, Millipore (Milan, Italy). All other chemical reagents were of analytical grade.

### 2.2. Caco-2 Cell Culture and Maintenance

Caco-2 cell line derived from human colorectal adenocarcinoma were cultured in vitro with complete DMEM culture medium (CM) (1% l-glutamine, 1% (*v*/*v*) antibiotic/antimycotic solution, and 10% (*v*/*v*) inactivated fetal bovine serum), in an incubator (Shellab^®^ Sheldon^®^ Manufacturing Inc., Cornelius, OR, USA) at 37 °C with 95% air and 5% CO_2_ atmosphere. At 80–90% of cell confluence, trypsinization was performed and the cell layer was harvested with CM. Afterward, the cell suspension was centrifuged (TC6, Sorvall Products, Newtown, CT, USA) at 1500 rpm for 10 min, eliminated the supernatant, and the cell pellet was re-suspended in 6 mL of CM. The number of cells in suspension was determined in a counting chamber (Hycor Biomedical, Garden Grove, CA, USA), using a 0.5% (*w*/*v*) trypan blue solution to visualize and count viable cells.

### 2.3. Preparation of ICG-Loaded NPs

ICG-loaded NPs were prepared using an emulsion polymerization method partially modified [3]. An appropriate amount of ICG (1 or 2 mg, ICG-NPs1, and ICG-NPs2, respectively) was dissolved into the polymerization medium, which consisted of 10 mL of an aqueous solution of hydrochloric acid (pH 2.5) containing Tween 20 (1.0% *w*/*v*). Subsequently, the ECA (1.0% *v*/*v*) was dropped into the reaction medium under continuous magnetic stirring, which was maintained until the polymerization was complete (3 h). Subsequently, the pH of the mixture was adjusted to 7.4 with NaOH 0.2 M to ensure the end of the polymerization reaction. All the reagents in excess, as well as free ICG, were removed using an ultrafiltration procedure on Amicon^®^ devices (30,000 Da MWCO) (Merk Millipore Ltd, Cork, Ireland). The volume of NP dispersion was concentrated to 1 mL, then 10 mL of bi-distilled water was added and the ultrafiltration was carried out. This ultrafiltration process was repeated five times. Once obtained, the purified NP suspension was collected in polyethylene containers and stored at 4 °C.

In order to check free ICG, all the filtered solutions were observed to evaluate the possible green color and analyzed by fluorometric spectroscopy.

### 2.4. Physicochemical and Ultrastructural Characterization of ICG-Loaded NPs

#### 2.4.1. Dimensional Analysis

Particle size and PDI (polydispersity index) of suspensions (ICG-NPs1 and ICG-NPs2) were determined by photon correlation spectroscopy (PCS) using a Coulter Submicron Particle Sizer N5 (Beckman-Coulter Inc. Miami, FL, USA). Before each measurement, the samples were diluted with bi-distilled water to obtain the concentration required by the equipment (range 5 × 10^4^ and 1 × 10^6^ counts s^−1^). Three samples of NP suspensions were prepared. Each sample was analyzed three times (*n* = 9) and results are expressed as mean ± standard deviation (SD).

#### 2.4.2. Evaluation of Physical Stability

Based on the obtained results, the formulation ICG-NPs1 was selected for further experiments. The physical stability of ICG-NPs1 was determined to evaluate if significant changes in particle size and PDI occurred over time. After preparation, the sample was stored at room temperature (25 °C) and 4 °C and dimensional analyses were performed after 7, 15, and 30 days, comparing the results with those obtained at the time of preparation.

#### 2.4.3. Ultrastructural Analysis (TEM) of ICG-NPs1

Morphological analysis of ICG-NPs1 ultrastructural features was conducted by TEM. For TEM observations, the procedure reported in Obinu et al. was followed [3]. Briefly, a drop (15 µL) of NP emulsion was deposited onto carbon-coated copper grids (200 mesh, Baltec) and, after 15 min, gently dried with a Whatman paper (Whatman^®^ Cellulose Filter Paper, Sigma-Aldrich, St. Louis, MO, USA) disk; then, a phosphotungstic acid drop was added and the sample was examined at the electron microscope as negatively stained structure [34,35].

JEOL JEM-1200 EX II microscope (JEOL ltd, Tokyo, Japan) operating at 100 kV (tungsten filament gun) and equipped with the TEM CCD camera Olympus Mega View III (Olympus Corp., Tokio, Japan) was used to obtain images at different magnifications (12,000× to 100,000×). The optical values of NPs were measured using ImageJ software ver. 1.49h (Waine Rasband; NIH, Bethesda, MD, USA) [36]. A semi-quantitative evaluation was performed on the acquired ultrastructural images (reference area 8.33 µm^2^), counting the number of ICG-loaded NPs.

#### 2.4.4. Fluorescence Studies

Fluorescence measurements were performed using an RF-6000 spectrofluorometer (Shimadzu, Kyoto, Japan) with an excitation wavelength of 785 nm. Emission spectra were recorded from 500 to 900 nm wavelength range. To evaluate the influence of ICG concentration on fluorescence intensity, the ICG-NPs1 dispersion and an aqueous solution of free ICG, containing the same concentration of dye (100 µg/mL), were diluted with bi-distilled water to obtain a series of samples with different ICG concentrations (range 0.1–100 µg/mL). The samples were analyzed and both the emission peak wavelength and the peak fluorescence intensity were recorded. Furthermore, unloaded NPs (prepared using the method described in Paragraph 2.3, without ICG) were examined to assess any interference in the emission spectrum.

In order to evaluate ICG fluorescence stability in water, an aqueous dispersion of ICG-NPs1 and an aqueous solution of free ICG, having the same concentration of ICG (10 µg/mL), were placed in the dark at 4 °C for up to 30 days. The ICG concentration was chosen on the basis of the results obtained in the experiment reported above. Fluorescence measurements were recorded at 0, 1, 2, 3, 4, 7, 10, 20, and 30 days and the time-dependent decay of ICG fluorescence was investigated and expressed as a percentage (%) of fluorescence remaining. Each obtained value is the average result of three experimental determinations.

### 2.5. Cytotoxicity Test

ICG-loaded NP cytotoxicity was investigated by MTT assay analyzing the effects of different NPs concentrations (750, 75, and 7.5 µg/mL of PECA, corresponding to ICG concentration 10, 1, and 0.1 µg/mL, respectively) on the viability of Caco-2 cells. Cells were seeded on 96-well plates (2.0 × 10^4^ cells in 200 μL of CM/well) and incubated at 37 °C and 5% CO_2_ for 24 h to reach subconfluence growth condition. ICG-loaded NPs were prepared in sterile bi-distilled water and diluted in CM and each sample (200 μL) was put in contact with cells for 24 h. CM alone was used as a control reference. After 24 h of contact with samples, cells were washed with 100 µL of PBS buffer (pH 7.4) and then put in contact for 3 h (37 °C and 5% CO_2_) with 50 μL of MTT at 7.5 μM concentration in 100 μL of DMEM without phenol red. Finally, 100 µL of DMSO was added to each well to allow the complete dissolution of blue formazan crystals, produced from the reduction of tetrazolium salt MTT by mitochondrial dehydrogenase of only viable cells. Solution absorbance was determined at 570 nm, with a 690 nm reference wavelength, employing an IMark1 Microplate reader (Bio-Rad Laboratories S.r.l., Segrate, Milan, Italy). Cell viability was expressed as a percentage calculated by normalizing the absorbance measured after contact with samples with those measured after contact with pure CM and used as positive control. For each sample were performed eight replicates.

### 2.6. Microscopic and Ultrastructural Analysis of Interactions between ICG-Loaded NPs and Caco-2 Cell Culture In Vitro

The interaction between ICG-NPs1 and cancer cells was investigated by TEM, incubating for 1 h the NPs with Caco-2 cells seeded in a multiwell plate (Corning^®^Costar^®^, Sigma-Aldrich, St. Louis, MO, USA). Samples without NPs were considered as untreated cell controls. ICG-loaded NPs were diluted in medium with ratio 1:1000 (0.1 µg/mL final ICG concentration). Cells were put in contact for 1 h with unloaded NPs and ICG-NPs1, washed, and fixed with a solution containing 2.5% glutaraldehyde and 2% paraformaldehyde in 0.1 M Na-cacodylate buffer (pH 7.4) for 6 h at 4 °C. Postfixation was performed using osmium tetroxide 1.33% in 0.1 M collidine buffer for 20 min at room temperature, then at 4 °C overnight. Cell specimens inside the plastic Petri were embedded in Epon resin cylinders (at 60 °C for 48 h) and subsequently scratched from the plastic support. Semithin sections (0.2 µm) for light microscopy stained with toluidine blue were observed with the Zeiss Axiophot microscope (Carl Zeiss, Oberkochen, Germany) provided with high-resolution optics, particularly an oil-immersion apochromatic objective with a magnification of 63× and a high aperture of 1.4. Images were recorded by 5.0 Mpxl CCD digital camera (Nikon corp., Tokyo, Japan), using a yellow filter to better identify the ICG content. Ultrathin sections (50 nm) for ultrastructural analysis were obtained by Ultracut Reichert (Reichert, Wien, Austria) provided with a DiATOME diamond knife and contrasted with uranyl acetate solution and lead citrate. To evaluate any interference, non-treated cells were used as control; furthermore, the uptake of unloaded NPs was assessed.

### 2.7. Statistical Analysis

Statistical analysis was performed with GraphPad Prism 5.0 software (GraphPad Software, Inc., San Diego, CA, USA). Data were analyzed using an unpaired *t*-test and the analysis of variance (one-way ANOVA) was followed by Tukey’s multiple comparison test. The significance level was set at *p* < 0.05.

## 3. Results

### 3.1. Preparation of ICG-Loaded NPs

An ultrafiltration procedure on Amicon^®^ devices (30,000 Da MWCO) has been carried out to control the free ICG. Due to its high water solubility, colored filtered solution will be expected. As observed results, all the filtered solutions were always uncolored and clear, indicating that there was not free ICG. In addition, no fluorescence was detected by fluorimetric analysis of the filtered solutions. On the basis of these results, we considered the encapsulation efficiency of the purified NP suspension of 100%.

### 3.2. Physicochemical and Ultrastructural Characterization of ICG-Loaded NPs

#### 3.2.1. Dimensional Analysis

The particle size of ICG-loaded NPs ranged from 120 to 200 nm and was influenced by the concentration of dye (Table 1). The mean diameter increased with the increase of ICG concentration (*p* < 0.05). Moreover, the PDI increased with ICG concentration (*p* < 0.05), indicating a reduction of suspension homogeneity. On the basis of these results, the formulation ICG-NPs1 was chosen for further studies.

#### 3.2.2. Evaluation of Physical Stability

**Figure 1:**
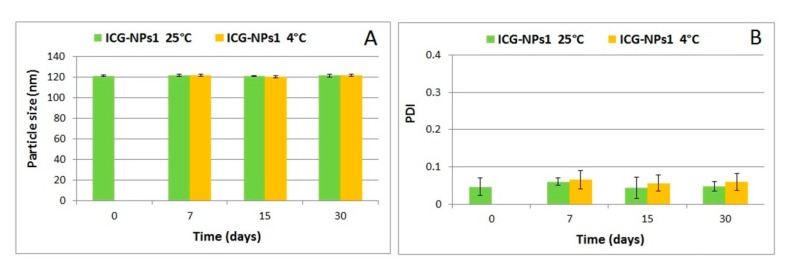
Physical stability of ICG-NPs1 in terms of particle size (**A**) and PDI (**B**).

The physical stability of ICG-NPs1 in terms of particle size and PDI as a function of time and storing conditions is shown in Figure 1.

The macroscopical observation of the NP suspensions confirmed that no visible aggregates of particles were present for the period of the study (one month). The dimensional analysis showed that NPs were dimensionally stable regardless of the storage temperature over time (*p* > 0.05). These results, although not performed in the conditions of a formal stability study, ensure that the sample was not modified during the subsequent studies on cell cultures.

#### 3.2.3. Ultrastructural Analysis (TEM) of ICG-NPs1

**Figure 2:**
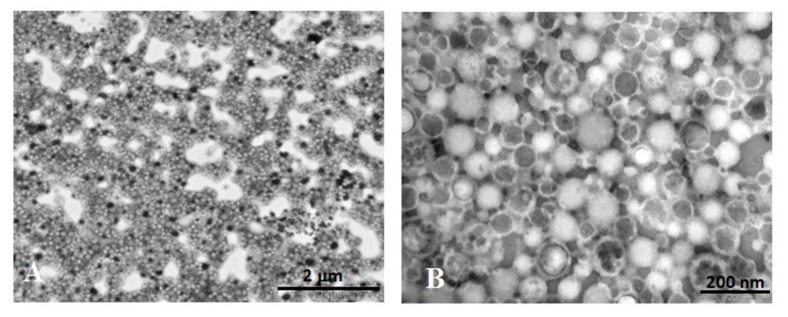
Ultrastructural morphology of negatively stained ICG-NPs1. (**A**) ICG-NPs1 appeared as aggregates creating a sort of network irregularly shaped (magnification: 12,000×; scale bar: 2 µm). (**B**) ICG-NPs1 appeared distinct in three different types for the morphology, dimension, and electron density (magnification: 100,000×, scale bar: 200 nm).

As indicated by the TEM images (Figure 2), ICG-NPs1 observed at low magnification (12,000×) (Figure 2A) showed a homogeneous spherical morphology. At higher magnification (100,000×), the NPs showed dimensions in line with the PCS analysis, with most of the NPs having a diameter of about 100 nm.

Mainly, three types of NPs different for the electron density distribution can be observed (Figure 2B). Some NPs showed a homogeneous and medium–low electron-dense content, extended to the whole NP observable area. The semiquantitative analysis indicated that this type represents the most frequent population (49.2% of the total number). Others (41.8%) showed a medium-high electron-dense content encircled by a thin and light peripheral ring. Neither of these two types of NPs present an ultrastructure similar to that of the unloaded PECA-NPs previously obtained [3]. Only a very small percentage of NPs (1.3%) appeared patchy due to the presence of lighter and variously shaped plots randomly distributed. 

#### 3.2.4. Fluorescence Studies

**Figure 3:**
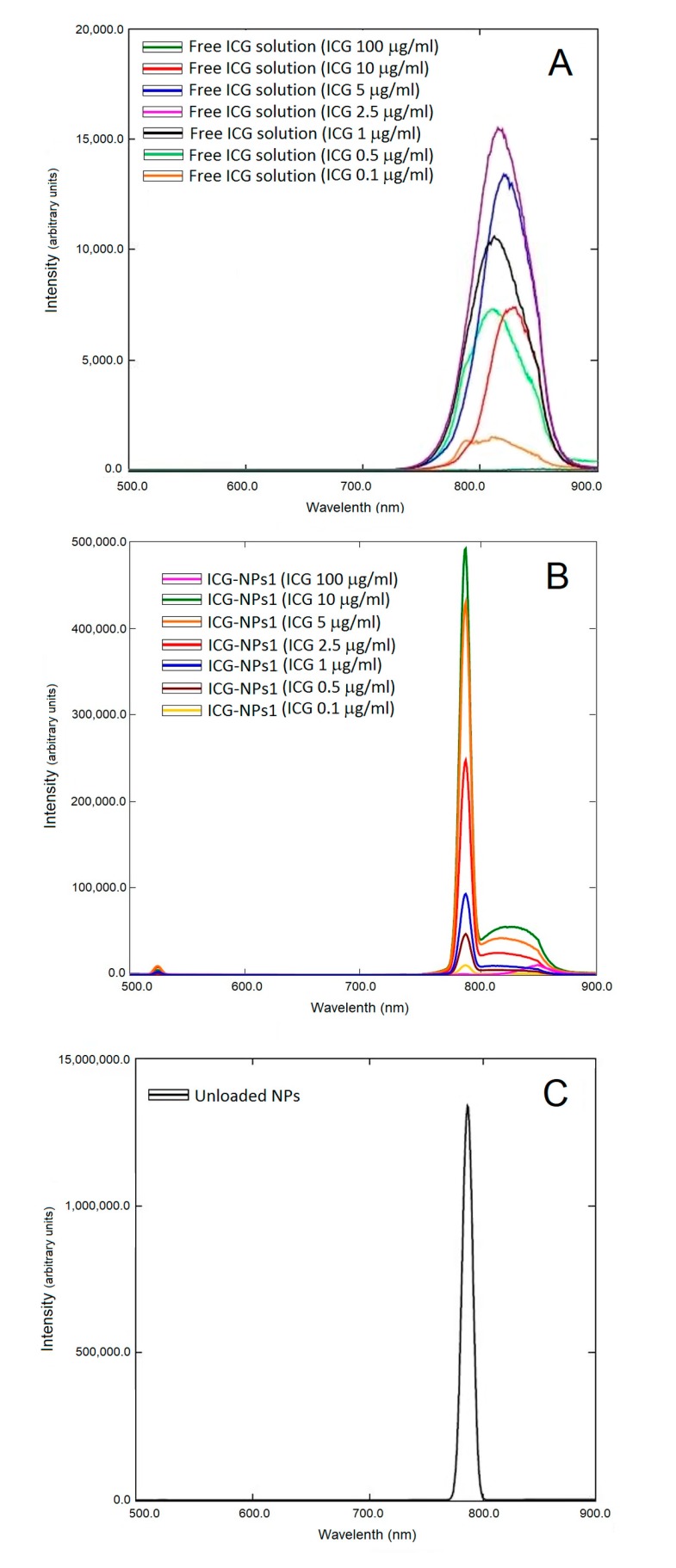
Fluorescence spectra of free indocyanine green (ICG) aqueous solutions (**A**), ICG-NPs1 aqueous dispersions (**B**) (ICG concentrations range 0.1–100 µg/mL), and unloaded nanoparticles (NPs) (**C**).

Fluorescence spectra of both ICG-NPs1 aqueous dispersions and free ICG aqueous solutions at different concentrations were reported in Figure 3. In the case of free ICG solutions (Figure 3A), the peak fluorescence intensity of ICG increased with an increase in dye concentration up to a maximum value of 2.5 µg/mL (fluorescence intensity, 15,528.6 a.u.); further increases in ICG concentration caused a gradual decrease in the peak fluorescence intensity. ICG-NPs1 dispersions (Figure 3B) followed almost the same trend, but the highest peak fluorescence intensity was found with an ICG concentration of 10 µg/mL (fluorescence intensity, 55,518.9 a.u.). A further increase in ICG concentration led to a decrease in peak fluorescence intensity. As the ICG concentration increased, the emission peak wavelength shifted from 810 to 854 nm for free ICG solutions, and from 812 to 850 nm for ICG-NPs1 dispersions. Moreover, fluorescence analysis of unloaded NPs (Figure 3C) confirmed that there were no interferences in the emission spectrum, as no fluorescence was found in the 800–850 nm emission wavelength range.

**Figure 4:**
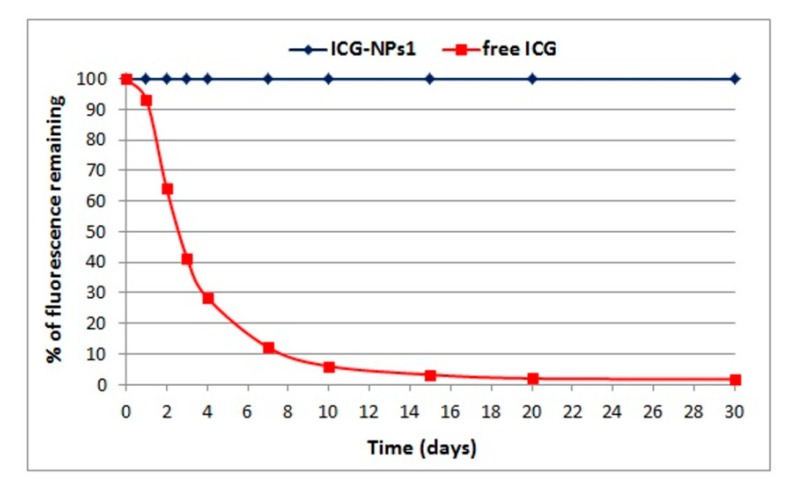
Fluorescence stability in water of free ICG solution and ICG-loaded NPs dispersion (ICG concentration, 10 µg/mL).

Based on the results reported above, the aqueous ICG-NPs1 suspension and the aqueous free ICG solution, containing an ICG concentration of 10 µg/mL, were chosen to evaluate the dye stability in water. Figure 4 shows the degradation pattern of free ICG solution and ICG-NPs1 dispersion over time. Interestingly, as evident from the results, fluorescence intensity values of ICG-NPs1 did not change during the time. On the contrary, a marked decrease in fluorescence intensity of free ICG was observed due to the degradation of dye in water.

### 3.3. Cytotoxicity Assay

**Figure 5:**
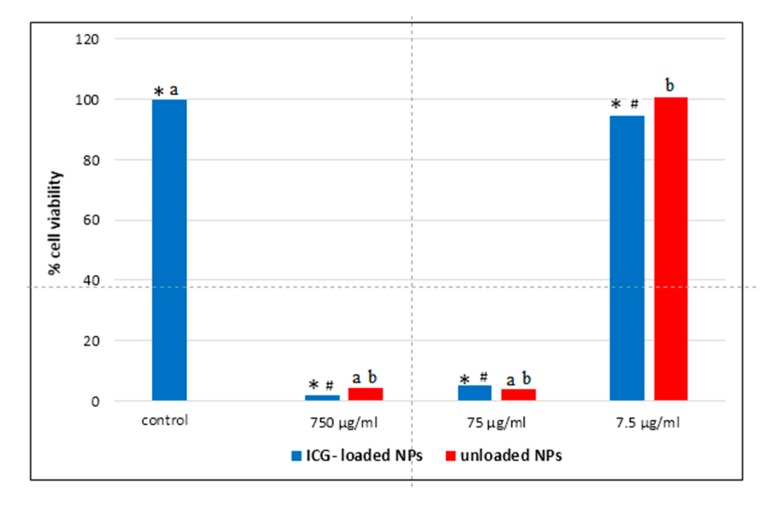
MTT test of ICG-loaded NPs and unloaded NPs against Caco-2 cells. ICG-loaded NPs: *p* < 0.05: * control vs. 750, 75, and 7.5 µg/mL; ^#^ 7.5 µg/mL vs. 750 and 75 µg/mL; unloaded NPs: *p* < 0.05: ^a^ control vs. 750 and 75 µg/mL; ^b^ 7.5 µg/mL vs. 750 and 75 µg/mL.

As displayed in Figure 5, Caco-2 cells treated for 24 h with NPs at concentration of 75 and 750 µg/mL (corresponding of 1 and 10 µg/mL of ICG, respectively) showed a decreased cell viability (compared to the control cells) to 5 ± 2.4% and 2 ± 1.6%, respectively (*p* < 0.05). Cells treated with NPs concentration of 7.5 µg/mL (ICG concentration of 0.1 µg/mL) presented less viability (94.3%) with respect to untreated Caco-2 cells used as a control (*p* < 0.05). However, if compared to samples with a concentration of 75 and 750 µg/mL, the NPs at the lowest concentration (7.5 µg/mL) led to reduced suppression of cell proliferation (*p* < 0.05), indicating dose-dependent cytotoxicity. ICG-loaded NPs at an ICG concentration of 0.1 µg/mL slightly affect Caco-2 cell viability, so this concentration was used for further experiments.

### 3.4. Microscopic and Ultrastructural Analysis of Interactions between ICG-Loaded NPs and Caco-2 Cell Culture In Vitro

**Figure 6:**
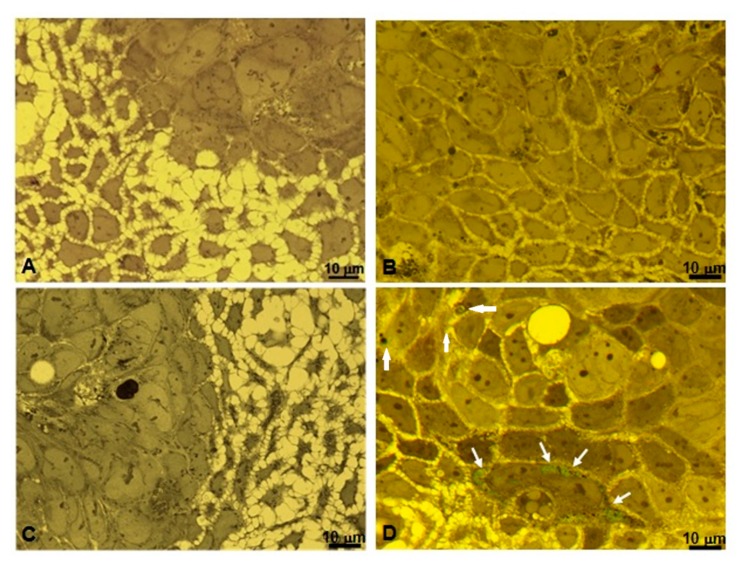
Semithin sections (0.2 µm) of human colorectal adenocarcinoma Caco-2 cell culture stained with toluidine blue and observed through yellow filter in the condenser stage of the light microscope. (**A**) Untreated cell culture (**B**) Cell culture treated with unloaded NPs. (**C**) Cell culture treated with free ICG in water solution. (**D**) Caco-2 cell culture treated with ICG-NPs1. Green spots are detectable inside cells (white arrows). All images were captured using an oil-immersion apochromatic objective 63x-high aperture 1.4 and each one shows a representative field of many cells. Scale bar: 10 µm.

Biological interaction in vitro between NPs and cells was studied at the light microscope on semithin sections stained with toluidine blue. Samples were observed through a yellow filter in the condenser stage of the light microscope in order to enhance the color contrast of ICG-loaded NPs. An untreated Caco-2 tumor cell line used as a control is represented in Figure 6A showing morphological features characteristic of this cell line in vitro. Both compacted and dispersed cells (not confluent growth) presented a normal cytoplasmic content and morphology, without any dots and green structures. Cells treated with unloaded NPs (Figure 6B) did not show any detectable NPs both inside and around the cells. Figure 6C represents cell culture treated with a free ICG solution. No presence of green dots or structures was detectable. Differently, Caco-2 cells treated with ICG-loaded NPs showed the presence of green small masses inside the cell cytoplasm (Figure 6D, white arrows).

**Figure 7:**
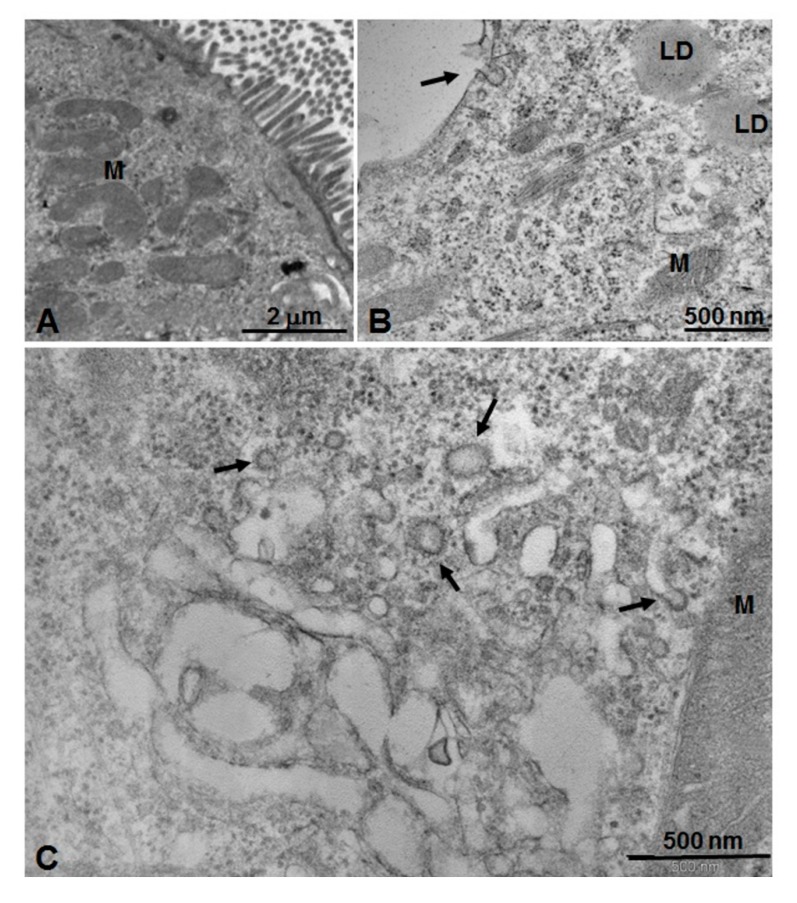
Electron micrographs (TEM) of Caco-2 cell grown in 2D culture in vitro stained with uranyl acetate and lead citrate, analyzed by a transmission electron microscope. (**A**) An untreated Caco-2 cell control with numerous microvilli on the apical surface (upper right) and mitochondria (M) inside the cytoplasm. Scale bar: 2 µm. (**B**) Superficial part of a cell with an evident caveola constituted by invagination of the plasma membrane-coated pits on its inner side (black arrow). In the cytoplasm are visible many free polyribosomes, bundles of cytoskeletal elements, some mitochondria (M), and lipid droplets (LD). Scale bar: 500 nm. (**C**) Cell treated with unloaded PECA-NPs. Numerous coated vesicles distributed in the peripheral part of the cytoplasm are present and associated with cytoplasmic membrane systems (arrows). Scale bar: 500 nm.

**Figure 8:**
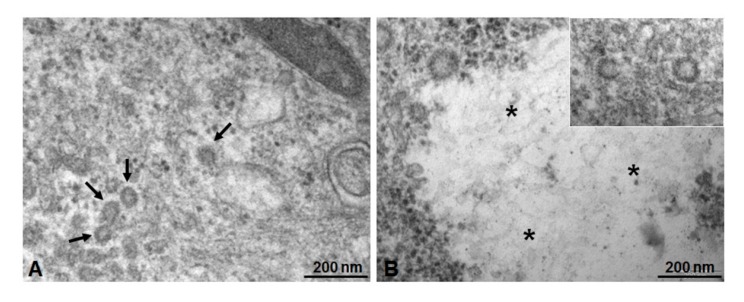
Electron micrographs (TEM) of Caco-2 cells treated with NPs and observed at the transmission electron microscope. (**A**) Cell culture with unloaded NPs, detectable as small rounded structures (about 100 nm sized) concentrated in cytoplasmic areas. Scale bar: 200 nm. (**B**) Caco-2 cell treated with ICG-loaded NPs. Rounded structures are distributed in cytoplasm (inset) and near to large light inclusion (asterisks). Scale bar: 200 nm.

Ultrathin sections for TEM of the same samples obtained by two-dimensional cell culture in vitro were stained with uranyl acetate and lead citrate (Figure 7 and Figure 8). Figure 7A,B show untreated Caco-2 cells (control). In particular, in Figure 7A, numerous microvilli can be seen on the external apical surface, forming the typical brush border associated with the absorbent activity of the enteric epithelium. In Figure 7B, it is possible to appreciate superficial caveolae constituted by pit-coated invaginations of the plasma membrane on their inner side (arrow). Many free polyribosomes, bundles of cytoskeletal elements, some mitochondria, and lipid droplets are also detectable in the cytoplasm (Figure 7B). After treatment with unloaded PECA-NPs (Figure 7C), numerous coated vesicles distributed in the peripheral part of the cytoplasm are present (Figure 7C, arrows).

In samples treated with ICG-loaded NPs (Figure 8), the deeper part of the cytoplasm appears rich in vesicles with a medium-high electron-dense content (Ø < 100 nm), often localized in small areas (Figure 8A, arrows). Figure 8B shows rounded structures distributed in the cytoplasm (inset), with homogeneous medium-low electron-dense content (asterisks). Inside it, a light and fine texture with constituting elements of about 100 nm can be recognized.

## 4. Discussion

ICG, a diagnostic fluorescent agent approved for clinical application, is usually used on cancer cells for tumor imaging [6,37] and can be combined with polymeric NPs to improve the effective functionality for imaging, therapy, and image-guided surgery [38]. PECA-NPs described in a recent paper [3] can be considered as a specific potential therapeutic platform for the treatment of solid tumors. For this reason, in the present study, ICG and PECA-NPs were associated to obtain a nanosystem, whose chemical-physical and morphological characteristics and behavior on tumor epithelial cell culture in vitro were evaluated. 

ICG-loaded NPs were prepared using an emulsion polymerization technique, a simple and reproducible process [3,39]. This method consists of a dropwise addition of the monomer into an HCl solution (pH < 3) containing a non-ionic surfactant, in our work the Tween 20 [39,40].

Dimensional analysis by PCS of ICG-loaded NPs showed two different classes of NPs, ranging from 120 (ICG-NPs1) to 200 nm (ICG-NPs2), maybe influenced by the concentration of dye, increasing the dimensions and reducing the homogeneity of suspension. On the basis of these results, further studies were conducted using the ICG-NPs1 formulation that showed for at least 1 month, both at room temperature and 4 °C, good physical stability in terms of dimensions and homogeneity of ICG dispersion. These results ensure a product that dimensionally corresponds to the original and consists of NPs that do not undergo aggregation during the time. As discussed below, changing in size could affect the clearance rate of NPs; moreover, dimensional stable NPs could be advantageous as they can be easily stored and used whenever necessary. Furthermore, the surgeon need not necessarily inject the NPs immediately before surgery.

In order to accumulate into tumor lesions by extravasation and enhanced permeability and retention effect (EPR), a nanosystem should be present in the bloodstream long enough to reach the site of action. However, the rapid clearance of circulating NPs is the major obstacle to realize these goals. As widely reported in several studies, particle size is a key factor in the biodistribution and blood circulation half-life of circulating NPs [41,42,43]. Small NPs (<10 nm) are subjected to tissue extravasation and renal clearance whereas large particles (>1 µm) are rapidly opsonized and eliminated from circulation via the RES or can accumulate in the liver and spleen. Therefore, polymeric NPs of intermediate size are ideal for in vivo applications because of their ability to circulate for a long time. These drug delivery systems are small enough to avoid opsonization and large enough to avoid renal clearance [44]. ICG-NPs1 showed hydrodynamic radii of 121.1 ± 0.75. It is an average value; indeed, the dimensional analysis revealed that in the dispersion were present different populations of NPs. In particular, the sample consisted of four NPs population with different particle sizes: 60 nm (16.3%), 112.9 (71.24%), 130.6 (12.08%), and 180.5 (0.38%). The blood clearance rate is directly correlated to the size of NPs; the larger NPs would be removed by the RES more quickly than smaller ones. As more than 85% of nanoparticles produced have a small size, the longer blood residence time and therefore the prolonged plasmatic half-life of included ICG would be expected. Besides particle size, other factors such as charge and shape influence the biodistribution profile of NPs. As argued below, TEM analysis showed spherical systems, indicating that the loading process did not change the shape of NPs compared to the unloaded NPs previously described [3]. These may have a positive effect on NPs uptake; indeed, as reported in our previous work, spherical-shaped particles have a higher probability of entering the cancer cells [3]. Surface charge also plays an important role in cellular accumulation; several research papers described how a negative charge can improve the NPs uptake in non-phagocytic cells such as Caco-2 cells [45,46]. Unloaded NPs [3] were characterized by a surface negative electrical charge; furthermore, literature data show that polymeric nanosystems encapsulating ICG had a negative zeta potential [47,48]. Therefore, a negative surface charge would be expected for ICG-loaded NPs, suggesting a suitable device to internalization inside the cells.

As described in the Results section, ICG-NPs1 observed at TEM with negative staining appear as spherical structures distinguishable in three types, depending on the electron density of the inner content. Due to the negative staining technique [34,35], their detection is due to the deposition of phosphotungstic acid (the electron-dense staining agent) into the interstitial space between NPs, giving to these a negative aspect. On the basis of this, only in a little percentage of NPs (1.3%), the content appears patchy due to some electron-transparent plots inside. These can be attributed to the unloaded NPs, as already discussed in our previous work [3] where large diffusion of the staining agent inside the unloaded NPs occurred. On the other hand, the electron transparency of the most of NPs (49.2%), due to the low molecular weight of the constitutive elements, gives a clear appearance in the electron image; anyway, the images suggest for these NPs a quite homogeneous structure. A second NP fraction (41.8%) shows a medium-high electron-dense homogeneous content inside, surrounded by a light peripheral ring suggesting a core-shell structure. The different features observed may be due to a whole homogeneous content for NP constituents for the second type, and to different degrees of diffusion of the negative stain agent inside NPs for the other NP ultrastructures, which could be attributed to a dissimilar distribution of ICG into the polymeric matrix.

The results obtained by fluorescence studies match well with those reported in the literature for an aqueous solution of ICG [7]. The increase in ICG concentration led to a growth in the peak fluorescence intensity up to a maximum value of dye amount of 2.5 µg/mL. This was followed by a decrease in the peak fluorescence intensity when the dye concentration is increased. Furthermore, as the ICG concentration increased, a shift in the emission peak wavelength was found. As previously hypothesized by Saxena et al., this shift may be because at high concentrations, ICG can form molecular aggregates that absorb and emit at longer wavelengths than the single ICG molecule [7]. The strong and sharp peak observed in Figure 3C was due to the effect of unloaded NPs scattering the excitation light. This peak was also observed in Figure 3B which reports fluorescence spectra of ICG-NPs1 at different dye concentrations. The high peak at 785 nm was due to unloaded NPs scattering whereas the peaks in the 800–850 nm wavelength range were due to ICG emission. The scattering band is much narrower than a typical fluorescence emission band. Consequently. if the excitation wavelength is changed, the scattering from the NPs will also shift and may interfere with the detection during surgery. However, as reported in the literature [17,49], during fluorescence-guided surgery, ICG fluorescence is detected with a near-infrared camera system, the Photodynamic Eye; as only emissions over 820 nm are detected, the peak at 785 nm does not interfere during the intraoperative visualization of ICG.

Fluorescence intensity values of ICG-NPs1 did not change during the time, showing an effective stabilization of ICG in the aqueous media when loaded into PECA-NPs. The increased stability of ICG-loaded NPs in water in comparison to free ICG aqueous solution is an interesting advantage in clinical application and it could be due to the interaction of the dye with the polymeric matrix, which avoids dye–dye interaction, favoring the presence of individual ICG molecules [50,51]. Indeed, as previously reported, the physicochemical transformations that ICG undergoes in aqueous media, such as aggregation, results in discoloration, reduced fluorescence, decreased light absorption, and shift of emission peak wavelength [7].

In order to study the cell interaction of ICG-loaded NPs, it has been utilized a Caco-2 cell line, widely used for pharmacological drug testing, as both an epithelial absorption barrier and a tumor cell model in vitro [52,53].

By comparing the cytotoxicity results of the ICG-loaded NPs and unloaded PECA-NPs previously studied [3], it is possible to argue that cytotoxicity is mainly due to the carrier and it is dose-dependent. Indeed, similar results were found when the Caco-2 cells were exposed to empty NPs correspondent to ICG-loaded NPs at 0.1, 1, and 10 µg/mL concentrations, indicating that the dye presence did not influence the cell viability. 

To highlight the interaction between cell culture and PECA-NPs unloaded and loaded with ICG, it was used a morphological approach by light and transmission electron microscopy [54,55]. Microscopic observations at a high resolution of epoxy resin embedded sections showed clearly that green cytoplasmic areas were present in Caco-2 cells treated with ICG-NPs1, while they could not be seen in untreated cells, in cells treated with unloaded NPs, and also in cell-cultured with ICG solution alone. This result suggests that the association of ICG to the NPs is determining for its internalization inside the cells, resulting in the concentration of ICG having evident green color inclusions. To better understand the possible mechanism of interaction between NPs and cells, the ultrastructure of these cell cultures after different treatments were studied by means of TEM analysis. In untreated cell control, coated pits vesicles on the plasma membrane were detectable, demonstrating endocytic activity. In cells treated with both unloaded and ICG-loaded NPs, specific relationships between coated vesicles and a particularly extended cytoplasmic membrane system suggested a complex dynamic mechanism, where endoplasmic reticulum and Golgi apparatus are realistically involved. Evidences of a specific pathway of NPs internalization through endocytosis are present; in particular, ICG-loaded NPs may be stored in internal cytoplasmic inclusions. In fact, the comparison between the observations of NPs isolated and negatively stained, and the observations of in vitro cultured cells, realistically leads to the following consideration: the green masses present in semithin sections of cultured cells after treatment with ICG-loaded NPs may be corresponding to the electron-transparent material observed in ultrathin section of electron micrography of the same sample. This could suggest the capture and internalization of ICG-loaded NPs through a mechanism mediated by clathrin-coated vesicles much more represented inside cells treated with ICG-loaded NPs. These considerations are in agreement with the recent literature showing various NPs (metallic, polymeric) internalized with a mechanism of clathrin-dependent endocytosis [55,56]. Moreover, further studies are mandatory for defining the specific uptake routes, using inhibitors of the different steps involved in internalization pathways [57,58].

Instrumental limitations to analysis and the complexity of pathways represent further issues, too. Further studies will be also conducted to better analyze the internalization and intracellular trafficking of these NP systems in tumor cells in vitro and ex vivo.

The future direction could be to specifically modify nanoparticle surface to improve uptake of the systems in tumoral cells, also by active intracellular transport. 

## 5. Conclusions

The results reported in the present research work demonstrated that the PECA-NPs previously proposed can be a promising carrier to load and stabilize ICG. In particular, the NPs here studied showed a particle size (<200 nm) suitable not only for the accumulation in tumor tissues by enhanced permeability and retention effect but also for endocytosis by tumor cells. In addition, the fluorescence studies confirmed that the spectra and the intensity values of ICG-loaded NPs did not change during the time, confirming a clear improvement of the stabilization of ICG in the aqueous media when incorporated into NPs. The possibility to prolong ICG-loaded NPs visualization could be useful during the surgical resection of the tumor. The antitumor activity previously described for PECA-NPs could be moreover exploited, maybe as a synergic effect of loaded anticancer drugs, to support the surgical removal of tumor lesions. The interaction of PECA and ICG-loaded PECA nanosystems with the tumor cells, involving an endocytic mechanism of uptake that leads to their internalization, was here demonstrated. All these results suggested that ICG-loaded NPs could be proposed as theranostic agents for tumor diagnosis and treatment. Anyway, the next goal will be to evaluate the selectivity of these PECA-NPs loaded with ICG, considering specific tumor cellular models (i.e., hepatocellular carcinoma and renal metastases), and the corresponding healthy tissue control (cell line of the human liver) for comparative studies.

Further, in vivo studies are required to confirm the real potential of this formulation.

## Figures and Tables

**Table 1:** Particle size and PDI (polydispersity index) of ICG-NPs1 and ICG-NPs2. Results are expressed as mean ± SD.

Sample	Mean Diameter (nm) ± SD	PDI ± SD
ICG-NPs1	121.1 ± 0.8	0.047 ± 0.023
ICG-NPs2	193.9 ± 5.1 *	0.127 ± 0.062 ^#^

* *p* < 0.05 vs. ICG-NPs1; ^#^ *p* < 0.05 vs. ICG-NPs1.
